# *In vitro* and *in vivo* characterization of Recifercept, a soluble fibroblast growth factor receptor 3, as treatment for achondroplasia

**DOI:** 10.1371/journal.pone.0244368

**Published:** 2020-12-28

**Authors:** Diogo Gonçalves, Guylène Rignol, Pierre Dellugat, Guido Hartmann, Stephanie Sarrazy Garcia, Jeffrey Stavenhagen, Luca Santarelli, Elvire Gouze, Christian Czech

**Affiliations:** 1 Research and Development, Rare Disease Unit, Pfizer, Nice, France; 2 TOLREMO Therapeutics AG, Muttenz, Switzerland; 3 Bionea, Biot, France; 4 Therini Bio, South San Francisco, CA, United States of America; 5 VectivBio, Basel, Switzerland; 6 Université Côte d’Azur, CNRS, Inserm, iBV, Nice, France; Universite de Nantes, FRANCE

## Abstract

Achondroplasia is a rare genetic disorder caused by mutations in the Fibroblast Growth Factor receptor 3 (FGFR3). These mutations lead to aberrant increase of inhibitory signaling in proliferating chondrocytes at the growth plate. Recifercept is a potential treatment for this disease using a decoy approach to sequester FGFR3 ligands subsequently normalizing activation of the mutated FGFR3 receptor. Recifercept binds to FGF isoforms *in vitro* and in cellular model systems and reduces FGFR3 signaling. In addition, in a transgenic mouse model of achondroplasia, Recifercept restores reduced body weight and long bone growth in these mice. These data suggest that Recifercept treatment could lead to clinical benefits in children treated with this molecule.

## Introduction

Mutations in the gene encoding fibroblast growth factor receptor 3 (FGFR3) are responsible for the phenotypes of several skeletal chondrodysplasias, including achondroplasia, the most common form of short limb and short stature. Achondroplasia is a rare genetic autosomal dominant disorder and has an incidence of 1:20’000 children. Children affected by achondroplasia suffer from abnormal long bone development, resulting in growth deficits [1]. In addition, they develop deformations of the skull and vertebrae leading to severe neurological and orthopedic complications.

The FGFR3 gene which codes for a tyrosine kinase coupled receptor, is located on the distal short arm of chromosome 4. In the early 90’s, different mutant alleles in FGFR3 were discovered that are causal for achondroplasia [2]. Most of the patients with the typical clinical features of achondroplasia have a spontaneous point mutation that results in a glycine to arginine substitution at amino acid 380 (G380R) located in the transmembrane domain of FGFR3 [3, 4]. FGFR3 normally functions to negatively regulate the differentiation and proliferation of chondrocytes to promote the normal endochondral growth of bones [4]. The G380R gain-of-function mutation prolongs the ligand-dependent activation of the FGFR3 tyrosine kinase activity leading to a constitutive activation of several downstream pathways required for normal bone growth.

FGFR3 is one of the four transmembrane FGF receptors (FGFR1-FGFR4) that contains an extracellular ligand binding domain, a transmembrane domain and two intracellular tyrosine kinase domains. The function of FGFR3 during skeletal development and postnatal growth was identified by analyzing the consequences of its mutation in mice. The global knockout of FGFR3 produces large mice with longer than normal limb bones [5]. In contrary, targeted overexpression of FGFR3 bearing the achondroplasia mutation to cartilage of transgenic mice produced a small mouse with short bones resembling those seen in human achondroplasia [6]. Similarly, cartilage targeted overexpression of the ligand FGF9 that activates FGFR3 generated a small mouse [7]. These different studies showed that altered regulation of FGFR3 by mutation or by genetic manipulation in mice produced a change in mouse size with impaired skeletal growth. Several strategies designed to reduce excessive FGFR3 signaling have been considered as possible treatment to normalize bone growth in achondroplasia, but as of today no approved cure is available [8]. Treatment with human growth hormone has been used but the success of this treatment is limited [9]. Vosoritide, a recombinant C-type natriuretic peptide (CNP) analogue, has been studied in a phase 2 trial and showed a sustained increase in the annualized growth velocity [10], however, no changes in other complications like skull development or proportionality of the limbs have been reported. Similar approaches are in clinical development with a modified CNP with longer half-life [11]. As symptomatic treatment, limb-lengthening surgery is used but this is a long and very invasive procedure [12]. In summary, there is still a high unmet medical need for an achondroplasia treatment.

Because activation of FGFR3 G380R requires ligand binding [13, 14], a possible treatment strategy was to use a soluble form of FGFR3 (sFGFR3) as a decoy receptor and titrate surrounding FGF especially, FGF9 and FGF18 which are known ligands of FGFR3 preventing their fixation on FGFR3 [15]. Based on the molecule published previously [15], we have developed and characterized a novel treatment paradigm suitable for treatment of children with achondroplasia.

Recifercept is currently in clinical development for treating achondroplasia in children. It is comprised of the extracellular domain of the FGFR3 and it is believed to act as a decoy receptor normalizing the aberrant signaling of the mutated human FGFR3 in achondroplasia. The proposed mechanism is sequestering extracellular FGF, inhibiting its binding to the mutated FGFR3 on chondrocytes and thereby reducing the increased signaling of the receptor in the growth plate promoting normal bone growth. Here, we show that Recifercept is binding with high affinity to FGF isoforms relevant for bone growth *in vitro* and cellular systems and that Recifercept is able to restore normal signaling in these cells. Moreover, treating a transgenic mouse model for achondroplasia [16] with Recifercept showed a dose-dependent increase in growth of long bones and body weight.

## Material and methods

### Surface plasmon resonance analysis of FGF–FGFR interactions

Recifercept–FGF interactions were characterized using a Biacore T200 instrument (GE Healthcare). Recifercept was immobilized on a CM5 sensor chip according to standard amine coupling protocol (GE Healthcare). Briefly, CM5 carboxymethyl groups were first activated using an injection pulse of 50 μl (flow rate, 10 μl/min) of an equimolar mix of N-ethyl-N-(dimethyaminopropyl)carbodiimide and N-hydroxysuccinimide (final concentration 0.05 M, mixed immediately prior to injection). Following activation, Recifercept was diluted to 5 μg/ml in 10 mM sodium acetate (pH 4) buffer and injected for 420sec with an aim ligand level of 400 RU. Excess unreacted sites were deactivated with a 40 μl injection of 1 M ethanolamine. The first flow cell (Fc) was used as a reference with a blank immobilization and Recifercept was immobilized on the second Fc to approximately 400 response units (RU). For kinetic, different concentrations of hFGF in HBS-EP buffer (0.01 M HEPES, 0.15 M NaCl, 3 mM EDTA, 0.05% polysorbate 20 (v/v), pH 7.4) were injected over the Recifercept CM5 chips at a flow rate of 50 μl/min. At the end of each sample injection (180 s), a 500 sec dissociation phase was performed with HBS-EP and the sensor surface was fully regenerated by injection of 50 μl of 100 mM sodium acetate, 2M NaCl (pH 4,5). Human FGF isoforms were analyzed by subfamily in order to run each subfamily on a new biosensor surface Each single cycle kinetic run was performed as follows, hFGF9 start run control + hFGFa, hFGFb etc, hFGF9 end run control. The FGF8 subfamily was analyzed with 16nM of Heparin in each tested concentration (Heparin sodium salt from porcine intestinal mucosa, Sigma).

### SPR data analysis

Reference responses from the control Fc (blank immobilization), were subtracted from Recifercept Fc for each analyte injection using BiaEvaluation software (GeHealthcare). The resulting sensorgrams were used for kinetic parameter determination by globally fitting the entire association and dissociation phases to a 1: 1 interaction. Five different analyte concentrations were used to determine the kinetic parameters for each interaction. Following curve fitting, each sensorgram was manually examined for data quality according the following acceptance criteria green quality control, reliable Rmax (not more than 10-fold the observed RU level response), Chi^2^ < 2 and U-value < 25. Detailed data are shown in Table 1 and S1 and S2 Figs.

**Table 1:** Recifercept-human FGFs kinetic constants.

	FGF1 subfamily		FGF9 subfamily			FGF8 subfamily*		
	hFGF1	hFGF2	hFGF9	hFGF16	hFGF20	hFGF8	hFGF17	hFGF18
K_on_ (1/Ms)	1.33E+06	2.79E+05	1.83E+06	2.80E+06	1.23E+05	1.71E+05	3.90E+05	2.70E+05
K_off_ (1/s)	9.04E-04	8.98E-04	1.33E-03	2.53E-03	5.48E-04	7.05E-04	9.64E-04	1.05E-03
**K**_**D**_** (nM)**	**0.74**	**3.4**	**0.75**	**0.94**	**7.1**	**4.28**	**2.73**	**4.17**
	FGF4 subfamily			FGF7 subfamily				
	hFGF4	hFGF5	hFGF6	hFGF3	hFGF7	hFGF10	hFGF22	
K_on_ (1/Ms)	NSB	NSB	NSB	NSB	NSB	NSB	NSB	
K_off_ (1/s)	NSB	NSB	NSB	NSB	NSB	NSB	NSB	
**K**_**D**_** (nM)**	**NSB**	**NSB**	**NSB**	**NSB**	**NSB**	**NSB**	**NSB**	

Binding between Recifercept and human FGFs isoforms was determined by surface plasmon resonance spectroscopy (SPR). Recifercept was immobilized on a CM5 chip and each FGF subfamily was analyzed on a new immobilized chip. hFGF9 was used as an internal run control and was loaded before and after the tested subfamilies. For each FGF, a single cycle kinetic was performed with 5 concentrations from 0 to 16 nM. Kinetic constants were measured on triplicate and K_ON_, K_OFF_ and K_D_ calculated from the mean value.

*FGF8 subfamily was analyzed with 16 nM of heparin in order to reach acceptance criteria. NSB refers to non-specific binding.

### Generation of BaF3-FGFR mutant cell lines

Murine pro-B lymphocyte BaF3 cells (DSMZ, Germany) were transduced with retroviral particles to stably overexpress either human FGFR3 IIIc or FGFR3 IIIc G380R. Briefly, GP-2 HEK packaging cell line (Clontech) was co-transfected with a pantropic VSVg vector (Clontech) and a pBABE-puro-hFGFR3 IIIc or hFGFR3 IIIc G380R (GeneArt, Thermo Fisher Scientific) using a standard CaCl2 procedure. After 48h, supernatant containing retroviral particles were harvested and viral RNA copy was determined by RT-qPCR (Retro-X™ qRT-PCR Titration Kit, Clontech).

BaF3 were then transduced by spinocculation (centrifugation 800g, 30 minutes at 32°C) and puromycin (Gibco) was added 3 days later to start the selection. 2μg/mL final puromycin concentration was chosen based on a puromycin kill curve made with non-transfected BaF3.

BaF3 mutant cells were cultured in RPMI-1640 medium (Gibco) supplemented with 10% heat inactivated fetal bovine serum (Gibco), 1% Penicillin/Streptomycin/Glutamine (Gibco), 10ng/mL recombinant murine IL-3 (R&D Systems) and 2μg/mL puromycin.

### BaF3 proliferation assay

5000 cells were plated in white 96-well plate with optical bottom (Nunc). Prior to plating, cells were centrifuged twice at 800g, 5 minutes at room temperature and washed with assay medium (RPMI-1640, 10% heat inactivated fetal bovine serum, 1% penicillin/streptomycin/glutamine) to remove of IL-3 and puromycin.

Cells were either stimulated for 72h with increasing concentrations of human FGF1 (Peprotech) or simultaneously with 10ng/mL human FGF1 and increasing concentrations of Recifercept, both supplemented with 10μg/mL heparin sodium salt (Sigma) acting as a co-factor. Cell proliferation was assessed by measuring intracellular ATP concentration with CellTiter Glo assay (Promega). Luminescence readout was measured with a Varioskan Lux plate reader (Thermo Fisher Scientific).

### RCS proliferation assay

Rat ChondroSarcoma cells (RCS) were kindly provided by Dr Claudio Basilico from New York University School of Medicine. Cells were maintained in DMEM high glucose (Gibco) supplemented with 10% hiFBS (Gibco) and 1% Penicillin/Streptomycin/Glutamine (Gibco). For proliferation assay, 2500 cells were plated in black 96-well plate with optical bottom (Nunc). 24h after plating, medium was removed and replaced by assay medium (DMEM high glucose, 1% heat inactivated fetal bovine serum, 1% Penicillin/Streptomycin/Glutamine). Cells were either stimulated for 48h with increasing concentrations of human FGF2 (Peprotech) or 0,3ng/mL human FGF2 and increasing concentrations of Recifercept, both supplemented with 1μg/mL heparin sodium salt. Cell proliferation was assessed by measuring cell nuclei number with CyQuant Direct assay (Thermofisher Scientific). Fluorescence was measured with a Varioskan Lux plate reader.

### Signaling experiment and western blot

RCS were plated at 100,000 cells in a 6-well plate in full medium. After 24h, medium was removed and replaced by assay medium. Cells were stimulated for 30 minutes with either 10ng/mL human FGF2 alone or in combination with 2μM Recifercept. Heparin sodium salt was added as a co-factor at 1μg/mL. Cells were washed twice in cold DPBS (Gibco) and lysed in RIPA buffer (Millipore) containing proteases and phosphatases cocktail inhibitors (Cell Signaling Technology). Cell lysates were centrifuged at 16,000g, 10 minutes at +4°C, and supernatants were harvested, and total protein concentration was measured by BCA assay (Thermofisher Scientific).

30μg protein under reducing conditions (DTT, +70°C, 10 minutes) were loaded on a Bis-Tris 4%-12% precast gel (Novex). After dry transfer, PVDF membrane (Novex) were blocked for 30 minutes and then incubated overnight at +4°C with the following rabbit antibodies (Cell Signaling Technology): phospho-PLCg1, PLCg1, phospho-MEK, MEK, phospho-ERK, ERK. All primary antibodies were used at 1:1000. Secondary anti-rabbit IgG-HRP (Cell Signaling Technology) was used at 1:5000 and WesternSure PREMIUM chemiluminescent substrate added. Images were taken with a C-Digit imager (Li-Cor). Phospho/Total protein ratios were calculated using Image 3.1. Software (Li-Cor)

### Recifercept drug substance

Recifercept is composed of the extracellular domain of the FGFR3 receptor (accession number P22607) from the amino acid 22 up to amino acid 358. There is an additional stretch of 13 amino acids added to C-terminus composed of the cytoplasmic domain of the FGFR3 from amino acid 423 up to amino acid 435. The full sequence of the molecule is: ESLGTEQRVVGRAAEVPGPEPGQQEQLVFGSGDAVELSCPPPGGGPMGPTVWVKDGTGLVPSERVLVGPQRLQVLNASHEDSGAYSCRQRLTQRVLCHFSVRVTDAPSSGDDEDGEDEAEDTGVDTGAPYWTRPERMDKKLLAVPAANTVRFRCPAAGNPTPSISWLKNGREFRGEHRIGGIKLRHQQWSLVMESVVPSDRGNYTCVVENKFGSIRQTYTLDVLERSPHRPILQAGLPANQTAVLGSDVEFHCKVYSDAQPHIQWLKHVEVNGSKVGPDGTPYVTVLKTAGANTTDKELEVLSLHNVTFEDAGEYTCLAGNSIGFSHHSAWLVVLPVSLESNASMSSNT.

For recombinant protein production the sequence was cloned in a eukaryotic expression vector.

For *in vitro* assays and the PK study, Recifercept recombinant drug material was generated from a 50L single use bioreactor (SUB) culturing from a GPEX CHO cell line. The media was G12.1, and the bioreactor was harvested on Day 14 at 81% viability. Media was filtered and drug substance was purified using chromatography.

The drug substance for the *in vivo* applications was generated using a 2L bioreactor culturing GPEX CHO cell line. The media was G12.1 and the bioreactor was harvested on Day 13 at 88% viability. Media was filtered and drug substance was purified using chromatography.

### Animals and housing

The Principles of Laboratory Animal Care (National Institutes of Health publication no. 85–23, revised 1985; http://grants1.nih.gov/grants/olaw/references/phspol.htm) and the European commission guidelines for the protection of animals used for scientific purposes (http://ec.europa.eu/environment/chemicals/lab_animals/legislation_en.htm) were followed at all times. All experiments have been approved by the national ethic committee (Ministère de l’Enseignement et de la Rercherche) under protocols APAFIS#l 5330–2018051717046781 v2 and APAFIS#20115–2019032211376608 v2 (PI: E Gouze).

Experiments were performed on male and female FVB transgenic Fgfr3^ach/+^ animals in which expression of the mutant FGFR3 is driven by the Col2a1 promoter/enhancer. Mice were exposed to a 12-hour light/dark cycle and had free access to standard laboratory food and water. Fgfr3^ach/+^ mice can develop complications of achondroplasia [15] therefore mice were observed daily with particular attention to locomotion and abdominal breathing. Animals found to have hemiplegia, paralysis indicated by bladder dysfunction, respiratory distress or continuing weight loss over three days were euthanized immediately. The rate of the survival in the WT group was 100% and the overall rate of the survival in the achondroplasia group was 67%.

At postnatal day 3 (PND3), all newborn mice from a single litter received the same dose subcutaneously (sc) with either vehicle control (PBS), Recifercept 3 mg/kg or 10 mg/kg twice weekly. Thereafter, sc injections were done at day 3, 5, 9, 11, 16, and 18. At PND3 77, 31 and 21 Fgfr3^ach/+^ animals were respectively reported in the PBS, 3mg/kg, and 10mg/kg group and 85, 37 and 26 WT animals were respectively reported in the PBS, 3mg/kg, and 10mg/kg group. The sample size was estimated from experience of previous experiment to be 10 litters per group [15]. At day 22, mice were sacrificed by intraperitoneal lethal injection of pentobarbital (100 mg/kg), skinned, and axial length measurements (from the nose to the end of the last caudal vertebra) were obtained using an electronic digital caliper. X-rays of all skeletons were taken with a Faxitron MX20 X-ray machine (Edimex) and the long bones (tibia, femur, ulna, humerus) were measured on the radiograph using Horos software. Genotypes were verified by polymerase chain reaction (PCR) of genomic DNA with the primers 5′- AGGTGGCCTTTGACACCTACCAGG-3’ and 5’- TCTGTTGTGTTTCCTCCCTGTTTGG-3’ which amplify 360 bp of the FGFR3 transgene.

### Pharmacology study in mice

In 6 weeks old FVB mice (Janvier Laboratory, France). Recifercept was administered once at 3 mg/kg or 10 mg/kg by intravenous (IV) injection. Total volume was supplemented with PBS 1X to inject 100μL per mouse. Blood was harvested at 10 min, 2h, 4h, 6h, 8h and 24h post drug administration. Recifercept levels in serum were measured by ELISA with MAB7662 (R&D Systems) as a coating antibody and a 7D7 custom detection antibody raised against a synthetic peptide of the D1 domain (KDGTGLVPSER).

### Statistical analysis

Statistical analyses were performed with GraphPad Prism 7.0 software (GraphPad Software Inc. San Diego, CA USA). To determine the statistical tests to be used, necessary assumptions were verified. To verify normality and equal variance, an Agostino and Pearson omnibus normality test (a = 0.05) and a Brown-Forsythe test (P < 0.05) were performed, respectively.

Because all skeletal measurement data sets (body weight, axial length, long bone length), fulfilled normality and equal variance requirements, two-tailed Student’s t test for comparisons of two independent groups was used in the different statistical analyses.

## Results

### Binding of Recifercept to different forms of FGF using surface plasmon resonance‎

Different recombinant human FGF isoforms have been used to analyze their interaction with Recifercept using Surface Plasmon Resonance‎ (SPR). Recifercept contains the full extracellular domain of the FGFR3 and the ligands FGF18, 9 and 16 are binding with high affinity to Recifercept (Table 1; S1 Fig; S1 Table). Moreover, also FGF1 and 2 have a strong affinity to Recifercept. In contrast, FGFs from the FGF4 and FGF7 subfamily do not show detectable binding to Recifercept using this methodology. In summary, the data show 0,74 nM and 3,4 nM affinity for the FGF1 subfamily, 7.1 nM to 0.75 nM affinity for the FGF9 subfamily and 4.28 nM to 2.73 nM affinity for the FGF8 subfamily (Table 1; S1 Fig; S1 Table). The FGF8 subfamily was tested by the addition of 16 nM heparin.

### Functional inhibition of FGF signaling in cells

**Fig 1:**
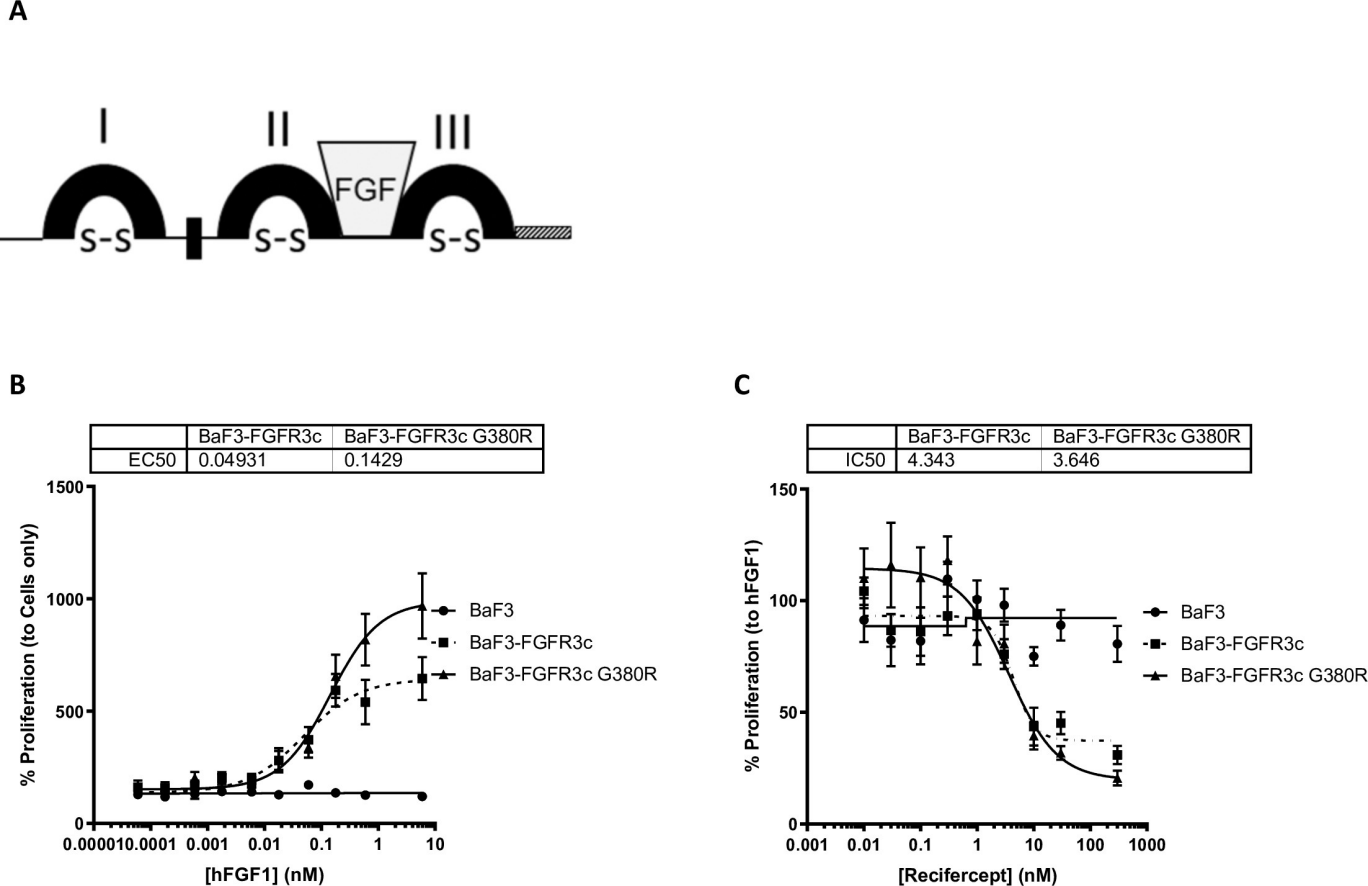
Recifercept inhibit hFGF1-mediated BaF3 mutant cell lines’ proliferation. Recifercept structure with 3 Ig domains (I, II, III), the FGF binding site (FGF), the acid box (black box), disulfide bridges (S-S) and the first 13 amino acid of the intracellular domain of FGFR3 (shaded box) (A). Control BaF3, BaF3 stably overexpressing either FGFR3 IIIc wt or FGFR3 IIIc G380R are stimulated with increasing concentrations of hFGF1 (nM) in presence of 10μg/mL heparin sodium salt and proliferation measured after 72h (B). These cell lines are then stimulated with 0,6nM hFGF1 and increasing concentrations (nM) of Recifercept in presence of 10μg/mL heparin sodium salt, and proliferation measured the same way (C). Graphs are representive of three independent experiments.

Recifercept is thought to act in part as a decoy to trap FGFs (Fig 1A). Trapped FGFs are unable to activate FGFR3, preventing downstream signaling from FGFR3 and blocking the negative growth signal of FGFR3 thereby resulting in increased cell proliferation.

In order to functionally test Recifercept, we worked with BaF3 overexpressing FGFR3. Pre-B murine lymphocyte BaF3 don’t express any of the four FGFR’s and can be easily transfected with either one or several receptors. These cells are very useful tools for drug screening purposes. Since we have shown that Recifercept binds to FGF1 with high affinity (Table 1), we stimulated the BaF3 cells stably transfected with either human FGFR3c WT or human FGFR3 G380R using different concentrations of FGF1 to measure proliferation. Fig 1B shows the increased proliferation in response to FGF1 in either WT or G380R transfected cells resulting in an EC50 of about 0.05nM and 0.14nM respectively. Non-transfected cells do not show any response upon stimulation with FGF1 arguing that this signal is due to the transfected FGFR3 (Fig 1B).

Next, to test if Recifercept was able to inhibit this proliferative signal, we tested FGF1 concentration of 10ng/ml (0.4nM) which gave a robust signal and added increasing concentrations of Recifercept [17]. The data show that Recifercept reduced proliferation in a dose-dependent manner with an IC50 of about 4.3nM in FGFR3 wt and 3.6nM FGFR3 G380R (Fig 1C).

**Fig 2:**
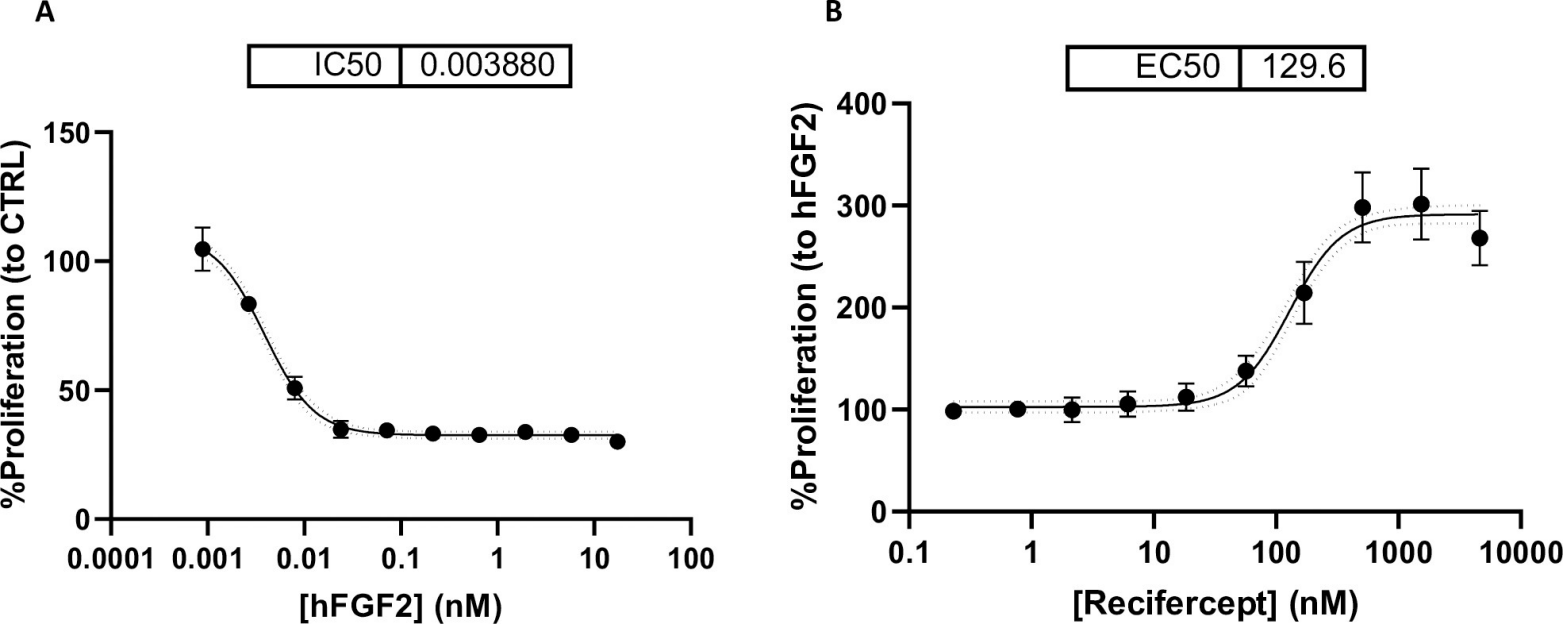
Reciferept inhibits hFGF2-driven effects and restores proliferation in RCS cells. RCS are stimulated with increasing concentrations (nM) of hFGF2 in presence of 1μg/mL heparin sodium salt, and proliferation is measured 48h after (A). RCS are stimulated simultaneously with 0,017nM hFGF2 and increasing concentrations of Recifercept (nM) in presence of 1μg/mL heparin sodium salt, and proliferation is measured after 48h (B).

Rat ChondroSarcoma cells (RCS) express different FGF receptors and in contrast to BAF3 reduce proliferation as in chondrocytes upon stimulation with FGF [18], (Fig 2A). In these cells, Recifercept restores the proliferation suppressed by FGF in a dose-dependent manner, with an EC50 of 129nM (Fig 2B).

**Fig 3:**
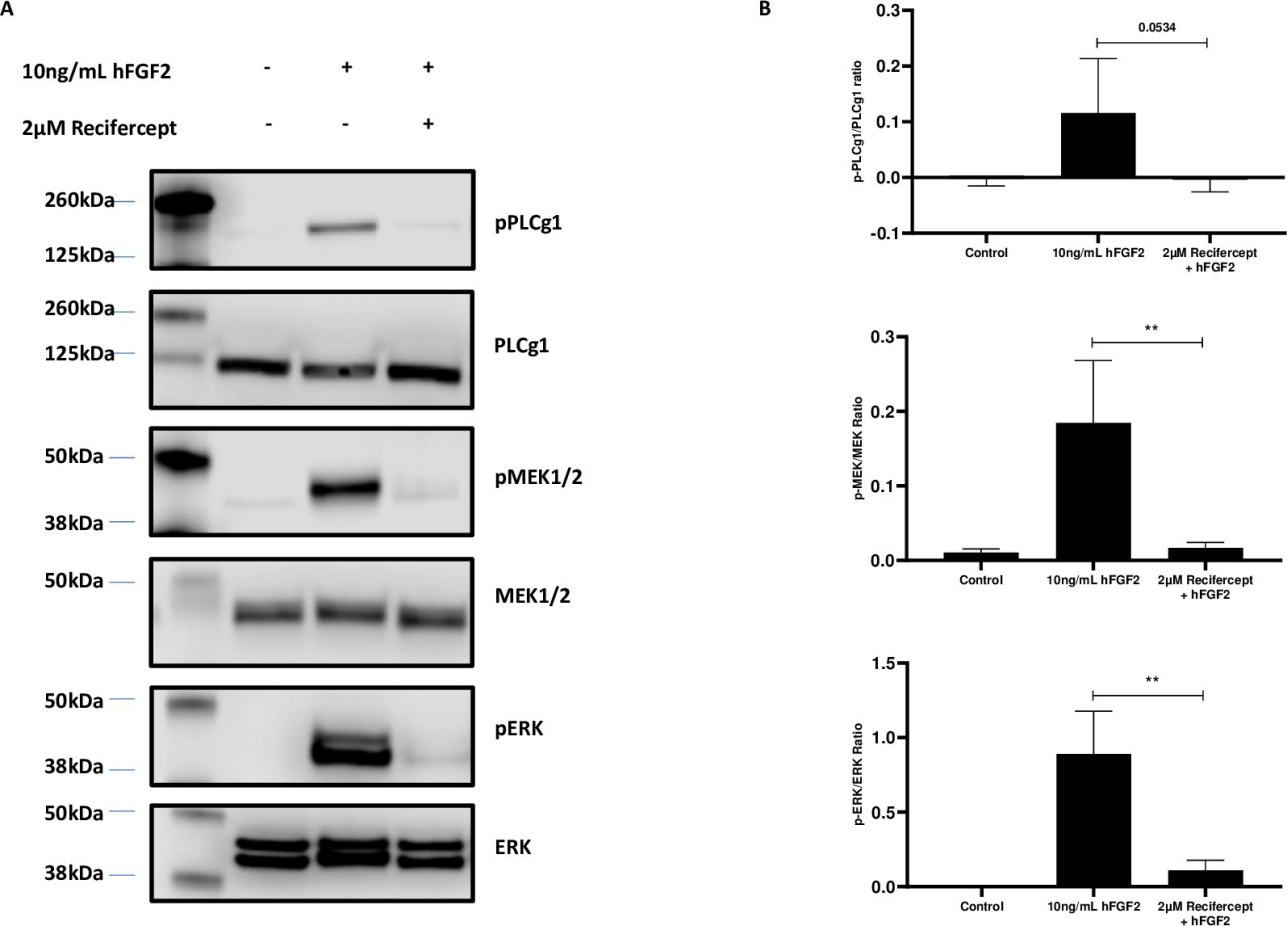
Recifercept can impair MAPK and pathway in RCS cells. RCS are either stimulated with 10ng/mL hFGF2 alone or in combination with 2μM Recifercept in presence of 1μg/mL heparin sodium salt for 30 minutes. Cells are then lysed and protein extracts are prepared under reducing conditions. 30μg are loaded on a gel, representative western blot of one experiment. (A). Phospho-total protein ratio is then measured. Data are representative of four independent experiments, negative values are an artefact of western blot densitometry. Statistics were done using un-paired t-test, **P < 0.01 (B).

We then analyzed the effect of Recifercept on signaling pathways downstream of FGFR3. The western blot in Fig 3A and 3B shows that upon FGF stimulation and Recifercept treatment two downstream signaling pathways are downregulated. The phosphorylated Phospholipase C and in addition the MAP-kinase pathway via pMEK1/2 and pERK in Western-blot are reduced, confirming a Recifercept inhibition of FGF downstream signaling. The MAP-kinase pathway is believed to be one of the main downstream pathway of FGFR3 signaling in chondrocytes [19]. These data together suggest that Recifercept reduces FGFR3 dependent signaling in cellular model systems.

### *In vivo* assessment of Recifercept pharmacokinetics and efficacy

**Fig 4:**
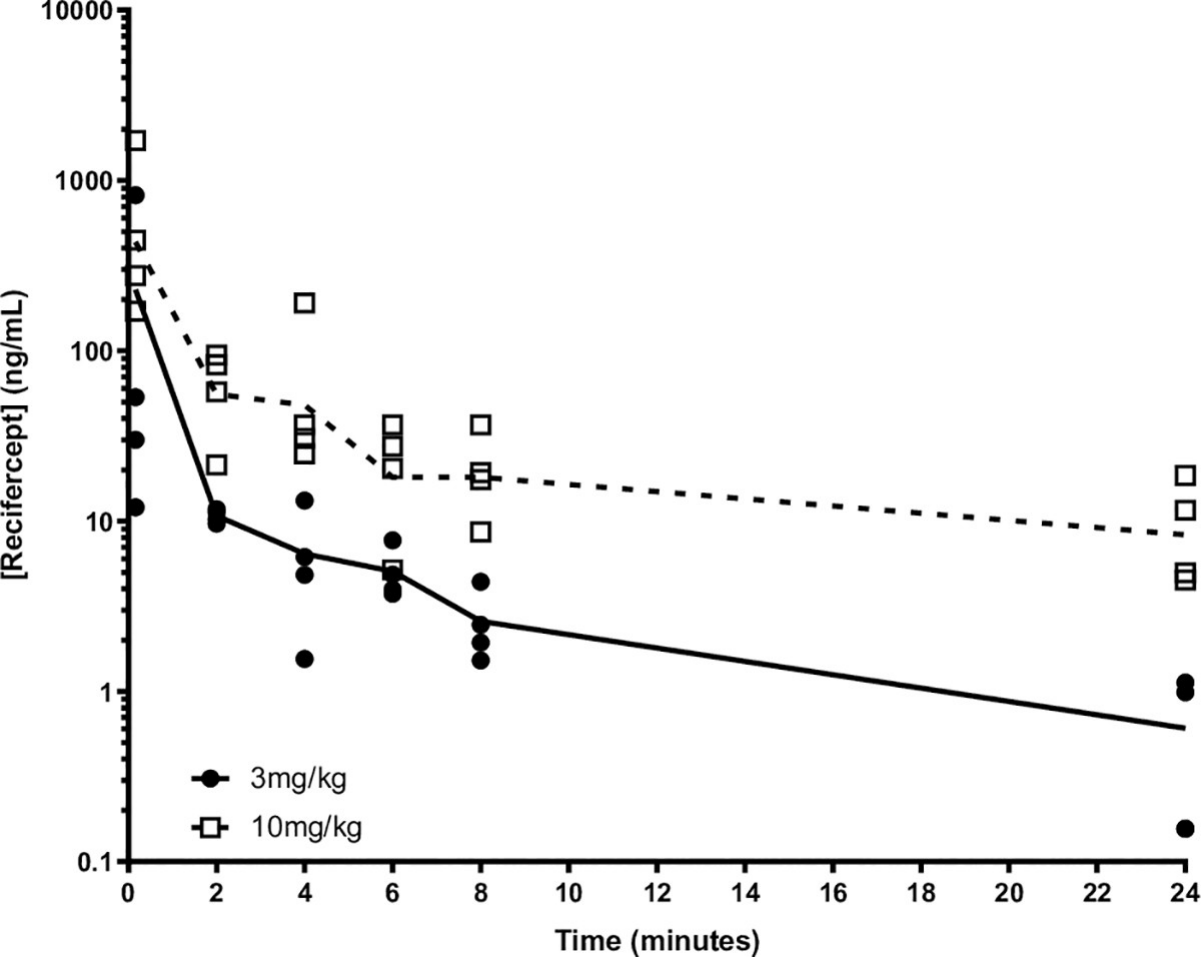
Pharmacokinetic analysis of Recifercept in WT mice. WT mice of 6 weeks of age were injected with Recifercept at 3 mg/kg or 10 mg/kg by intravenous injection (IV) in a single dose PK study using the following time points: 10 min, 2h, 4h, 6h (terminal), 8h (terminal), 24h (terminal) with two blood draws per mouse. Both doses show a typical IV profile with fast initial clearance from 10 min to the 2 hour time point and a slower terminal clearance as measured in the 2 hour to 24 hour time points.

To better understand the *in vivo* pharmacological properties of Recifercept, we dosed 6-week-old WT mice with a single intra-venous dose at 3mg/kg and 10mg/kg and analyzed the Recifercept exposure in serum after different timepoints post dose. Fig 4 shows the profile of Recifercept exposure in serum over 24h. Both doses show a fast-initial clearance from 10 min to the 2h and a slower terminal clearance and a half-life time of about 6h. The data show that for both doses, serum exposures sufficient for pharmacological activity *in vivo* can be reached.

**Fig 5:**
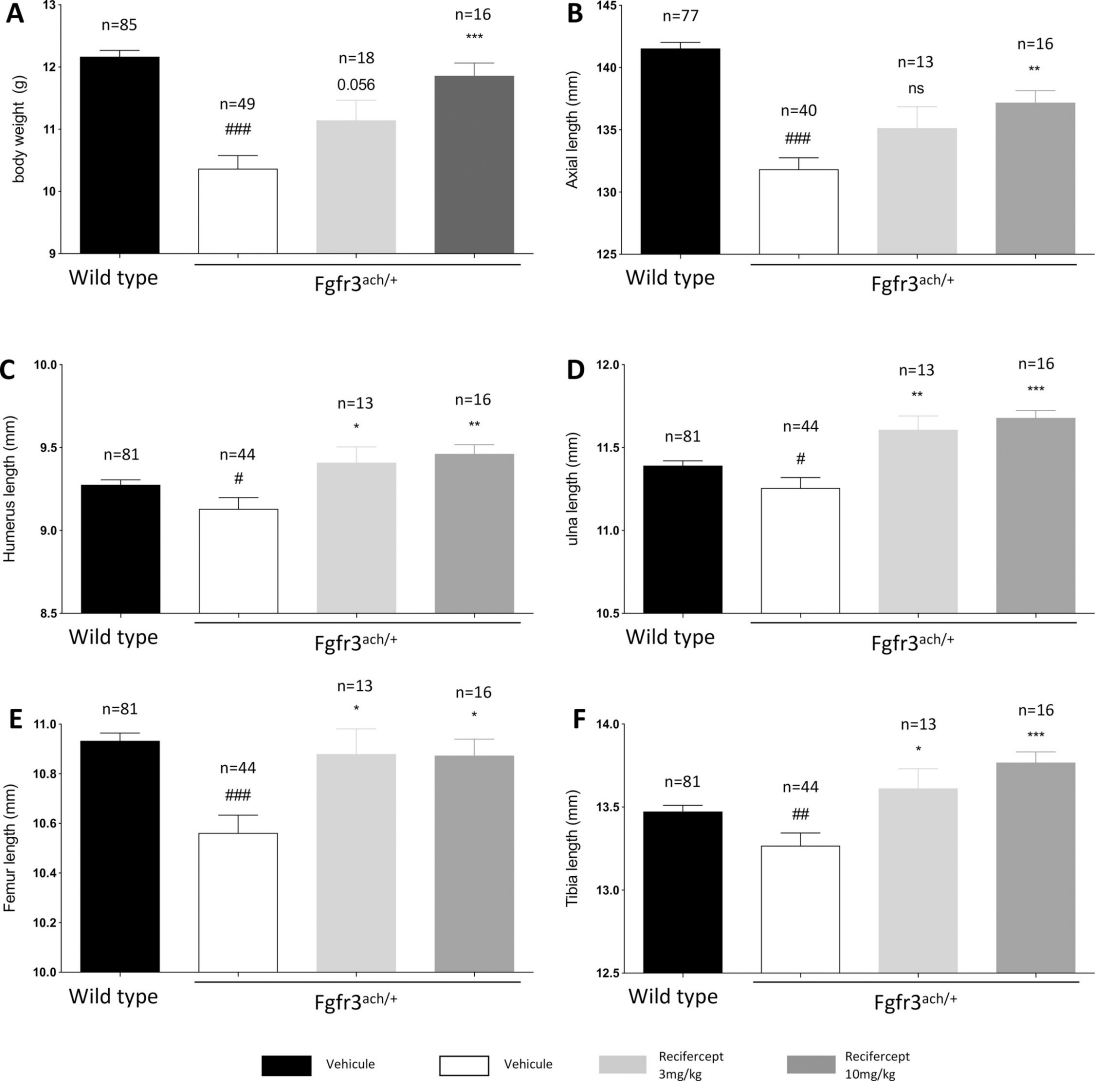
Recifercept treatment improves body weight, axial length and long bones length. WT and Fgfr3^ach/+^ mice received subcutaneous injection of vehicle or Recifercept at 3 or 10 mg/kg. Growth was characterized by body weight (A), total length (B), and long bones measurements (C-F). Numbers of mice in each group are indicated, due to technical reasons not all measurements could be taken of every mouse. Data followed normal distribution. *P < 0.05, **P < 0.01, ***P < 0.001 versus Fgfr3^ach/+^ vehicle-treated mice, # p< 0.05, ## p< 0.01, ### p< 0.001 versus WT vehicle-treated.

We have previously shown that the soluble form of the FGF receptor rescues several growth parameters seen on the transgenic Fgfr3^ach/+^ mice [15]. The Fgfr3^ach/+^ mice show pronounced deficits in body weight, axial length and length of the long bones (humerus, ulna, femur and tibia) at the age of 21 days (Fig 5). Since Recifercept is a close derivative of this molecule, we confirmed the *in vivo* activity of the molecule in the Fgfr3^ach/+^ mice using a similar treatment paradigm starting at PND3 and twice a week dosing with 3mg/kg and 10mg/kg for 21 days. The treatment was well tolerated by the mice without any overt signs of adverse events.

**Fig 6:**
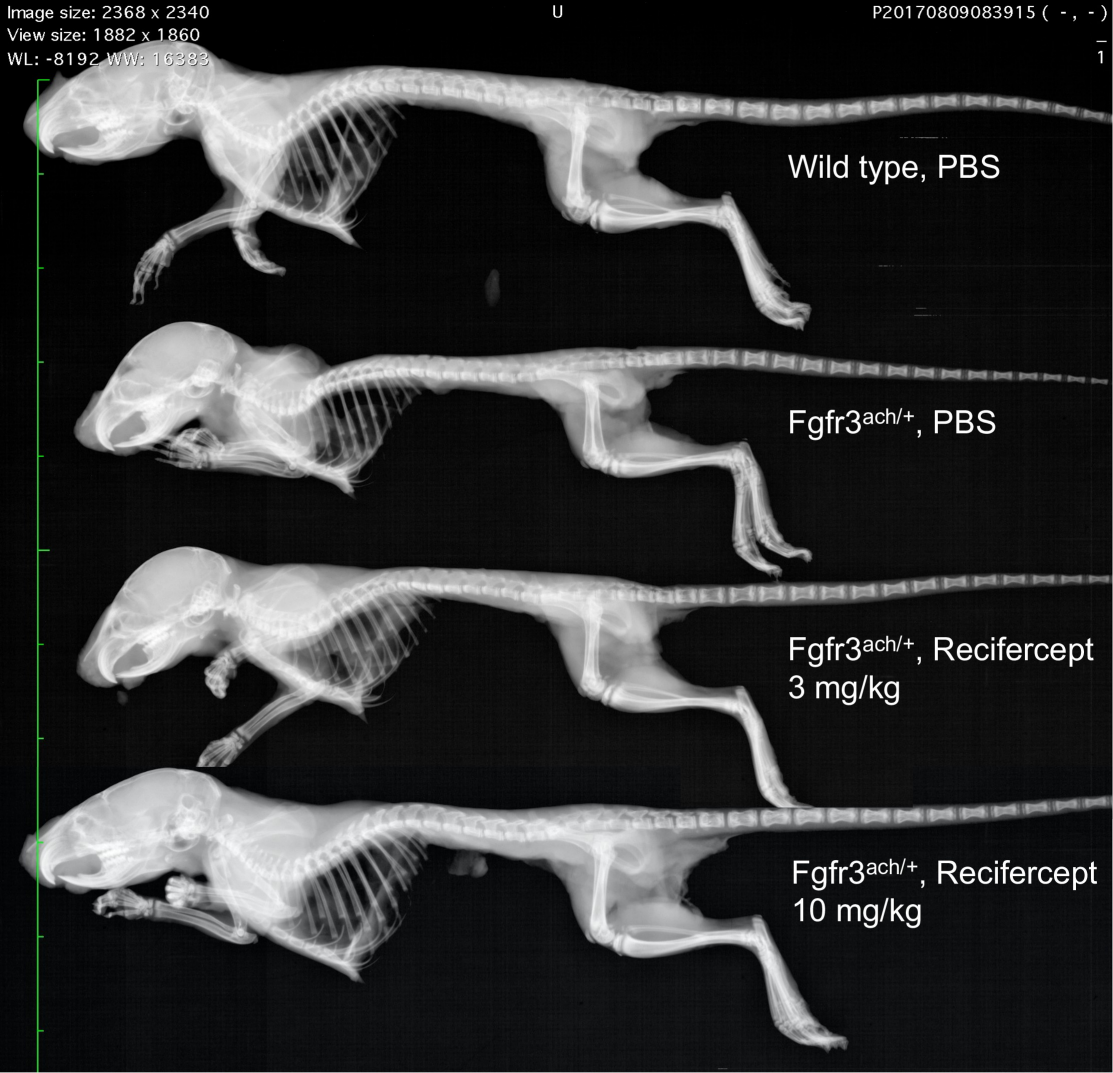
Recifercept treatment improves overall skeletal growth. Representative skeletons of PND22 WT and Fgfr3^ach/+^ mice that received subcutaneous injection of vehicle or Recifercept at 3 or 10 mg/kg. Digital X-Ray were recorded at 26 kV for 19 seconds using a Faxitron MX20 device.

The results are shown in Fig 5. Recifercept rescues the body weight deficits of the Fgfr3^ach/+^mice to almost normal levels at 10mg/kg (Fig 5A). Axial length is significantly increased for the 10mg/kg treatment group compared to vehicle (Figs 5B and 6). Moreover, Recifercept treatment had a dose-dependent effect on skeletal bone growth as seen in tibia, ulna, femur and humerus measurements compared to vehicle. The growth of this long bones reaches normal level already at 3mg/kg (Fig 5C–5F). These results show that Recifercept *in vitro* activity shown above in SPR and different cellular systems also translates into efficacy in an animal model.

## Discussion

Here we report the molecular characterization of Recifercept, a novel molecule in clinical development for achondroplasia. Recifercept is a modified soluble recombinant human FGFR3 designed to be a decoy protein, competing for ligands of the FGFR3-G380R receptor responsible for achondroplasia.

There are 22 known human FGFs, divided by mode of action, paracrine, endocrine and intracrine. The third subset of FGFs (FGF11-14) lack signal sequences and are thought to remain intracellular [20]. Paracrine FGFs include the FGF1 subfamily (FGF1 and FGF2), the FGF4 subfamily (FGF4, FGF5, and FGF6), the FGF7 subfamily (FGF3, FGF7, FGF10, and FGF22), the FGF8 subfamily (FGF8, FGF17, and FGF18), and the FGF9 subfamily (FGF9, FGF16, and FGF20). Endocrine FGFs are represented by the FGF19 subfamily (FGF19, FGF21, and FGF23) [21]. Most relevant ligands of this receptor for bone growth regulations are FGF18, FGF9 and FGF2, which have shown high affinity to Recifercept (Table 1).

Growth of long bones is regulated at the growth plate located at the ends of growing endochondral bones [22]. The growth plate consists of three highly organized layers of cells, the resting, proliferating and hypertrophic zones. The chondrocytes in the proliferating and hypertrophic zone are the key regulators of bone growth and the key signaling pathways are mediated through FGFR3. Following binding of FGFs, the receptors dimerize and initiate the signaling cascade [23]. Two main downstream signaling pathways are associated with FGF signaling and interestingly, these signals are inhibitory to bone growth [24]. First, the Ras/MAPK pathway propagates signals that negatively affect terminal differentiation and post-mitotic matrix synthesis and second the STAT1 (signal transducer and activator of transcription 1) pathway mediates the inhibition of chondrocyte proliferation [24]. Both pathways are activated in proliferating chondrocytes at the growth plate [25].

The data presented here show that Recifercept is binding *in vitro* with high affinity to several FGF isoforms using Surface Plasmon Resonance. Recifercept effectively binds human FGF2, human FGF9 and human FGF18 with nanomolar affinity. This high affinity interaction would prevent FGF from binding to its receptors and suggests, that Recifercept acts as a decoy to the FRGR3 receptor. This is further substantiated using several cellular assays.

In our cellular models, we have shown that Recifercept is influencing directly the pathways involved in regulation of chondrocyte proliferation. However, the exact mechanism of action cannot be deciphered from these experiments. As mentioned above, FGFR3 shows a ligand-dependent activation which involves receptor dimerization. In the model systems, we cannot clearly distinguish between a direct effect on the FRGR3 receptor by a possible formation of heterodimers inhibiting the activation and the decoy effects sequestering exogenous FGF2. Further studies will be needed to better understand the contribution of the different mechanisms to the *in vivo* activity of Recifercept.

The *in vitro* activity shown in SPR and different cellular models translates well into *in vivo* activity on bone growth in transgenic Fgfr3^ach/+^ mice. Recifercept corrects some of the key deficits of the Fgfr3^ach/+^ transgenic mice such as body weight and body length and growth of long skeletal bones.

Other molecules targeting bone growth signaling pathways have been reported to be successful in showing treatment effects in transgenic mice and also recently in clinical trials on a pediatric population. Vosoritide is a CNP analog acting on the natriuretic peptide receptor 2 (NRP2). This receptor is strongly expressed on chondrocytes and its downstream signaling also activates MAP-kinase pathway. However, the activation of the NRP2 pathway does not fully rescue the activation of FGFR3; specifically, STAT1 signaling is not activated by NRP2 [26]. Nevertheless, treatment of transgenic mice with achondroplasia also increased the growth of long bones [27] similarly as we have reported here and more recently, children with achondroplasia treated with different doses of Vosoritide in a clinical phase 2 study demonstrated increase in annualized growth velocity [10].

Together with our data presented here, this supports the approach to modulate cellular signaling on chondrocyte pathways to clinically benefit patients, and that efficacy on transgenic mice can translate into efficacy in patients.

## Conclusion

The data presented demonstrate that Recifercept is binding to FGF 1, 9 and 8 subfamilies *in vitro* and reverts the FGF dependent activation of FGFR3 in cellular models system. Moreover, in a transgenic Fgfr3^ach/+^ mice, dose-dependent treatment with Recifercept rescues the disease phenotype and restores skeletal growth. Taken together, these data strongly suggest that Recifercept, currently in clinical development, would be able to reverse the molecular mechanisms responsible for the observed growth deficits in children with achondroplasia.

## Supporting information

S1 Checklist The ARRIVE essential 10: Author checklist. (PDF)

S1 Table Recifercept-human FGFs kinetic constants raw data. Binding between Recifercept and human FGFs isoforms was determined by surface plasmon resonance spectroscopy (SPR). Recifercept was immobilized on a CM5 chip and each FGF subfamily was analyzed on a new immobilized chip. hFGF9 was used as an internal run control and was loaded before and after the tested subfamilies. For each FGF, a single cycle kinetic was performed with 5 concentrations from 0 to 16 nM. Following curve fitting, each sensorgram was manually examined for data quality according the following acceptance criteria green quality control, reliable Rmax (not more than 10-fold the observed RU level response), Chi^2^ < 2 and U-value < 25. hFGF9 was used as an internal run control and was loaded before and after the tested subfamilies. * FGF8 subfamily was analyzed with 16 nM of heparin in order to reach acceptance criteria.
(DOCX)

S1 Fig Recifercept-human FGFs sensorgrams. Binding between Recifercept and human FGFs isoforms was determined by surface plasmon resonance spectroscopy (SPR). Reference responses from the control Fc (blank immobilization), were subtracted from Recifercept Fc for each analyte injection. The resulting sensorgrams were used for kinetic parameter determination by globally fitting the entire association and dissociation phases to a 1: 1 interaction. hFGF9 was used as an internal run control and was loaded before and after the tested subfamilies. For each FGF, a single cycle kinetic was performed with 5 concentrations from 0 to 16 nM.
(TIF)

S2 Fig Western blot raw images. Western blots from Fig 3 were taken from these raw images. All lane marked with a “X” have not been taken into account for the present study.
(TIF)
