# Supplementary material for: In vitro and in vivo characterization of Recifercept, a soluble fibroblast growth factor receptor 3, as treatment for achondroplasia
Source: PLoS One. 2020 Dec 28;15(12):e0244368. doi: 10.1371/journal.pone.0244368 (PMC7769458; doi:10.1371/journal.pone.0244368)
Supplement: S1 Table — Binding between Recifercept and human FGFs isoforms was determined by surface plasmon resonance spectroscopy (SPR). Recifercept was immobilized on a CM5 chip and each FGF subfamily was analyzed on a new immobilized chip. hFGF9 was used as an internal run control and was loaded before and after the tested subfamilies. For each FGF, a single cycle kinetic was performed with 5 concentrations from 0 to 16 nM. Following curve fitting, each sensorgram was manually examined for data quality according the following acceptance criteria green quality control, reliable Rmax (not more than 10-fold the observed RU level response), Chi2 < 2 and U-value < 25. hFGF9 was used as an internal run control and was loaded before and after the tested subfamilies. * FGF8 subfamily was analyzed with 16 nM of heparin in order to reach acceptance criteria. (DOCX) [file pone.0244368.s002.docx]

**S1 Table. Recifercept-human FGFs Kinetic Constants Raw Data.**

Binding between Recifercept and human FGFs isoforms was determined by surface plasmon resonance spectroscopy (SPR). Recifercept was immobilized on a CM5 chip and each FGF subfamily was analyzed on a new immobilized chip. hFGF9 was used as an internal run control and was loaded before and after the tested subfamilies. For each FGF, a single cycle kinetic was performed with 5 concentrations from 0 to 16 nM. Following curve fitting, each sensorgram was manually examined for data quality according the following acceptance criteria green quality control, reliable Rmax (not more than 10-fold the observed RU level response), Chi^2^ < 2 and U-value < 25. hFGF9 was used as an internal run control and was loaded before and after the tested subfamilies. * FGF8 subfamily was analyzed with 16 nM of heparin in order to reach acceptance criteria.

|  |  | K_on_ (1/Ms) | K_off_ (1/s) | KD (nM) | R_max_ (RU) | χ^2^ (RU)^2^ | U-value |
| --- | --- | --- | --- | --- | --- | --- | --- |
| FGF1 | µ (n=3) | 1.33E+06 | 9.04E-04 | **0.74** | 14.5 | 0.8 | 3.3 |
|  | σ (n=3) | 4.44E+05 | 9.40E-05 | 0.3 | 12.9 | 1.2 | 0.6 |
| FGF2 | µ (n=3) | 2.79E+05 | 8.98E-04 | **3.4** | 36.7 | 1.77 | 3.7 |
|  | σ (n=3) | 7.26E+04 | 9.96E-05 | 1.02 | 4.2 | 0.28 | 1.2 |
| FGF9 | µ (n=3) | 1.83E+06 | 1.33E-03 | **0.75** | 44 | 1.38 | 2.3 |
|  | σ (n=3) | 2.08E+05 | 5.77E-05 | 0.12 | 4.2 | 0.58 | 1.5 |
| FGF16 | µ (n=3) | 2.80E+06 | 2.53E-03 | **0.94** | 10.8 | 0.76 | 3.7 |
|  | σ (n=3) | 5.26E+05 | 3.51E-04 | 0.31 | 2.1 | 0.45 | 0.6 |
| FGF20 | µ (n=3) | 1.23E+05 | 5.48E-04 | **7.1** | 24.3 | 0.1 | 1.5 |
|  | σ (n=3) | 1.05E+05 | 1.27E-05 | 5.11 | 10.9 | 0.12 | 0.7 |
| FGF8* | µ (n=3) | 1.71E+05 | 7.05E-04 | **4.28** | 61.7 | 0.31 | 1 |
|  | σ (n=3) | 4.47E+04 | 1.85E-04 | 1.48 | 28.9 | 0.09 | 0 |
| FGF17* | µ (n=3) | 3.90E+05 | 9.64E-04 | **2.73** | 23.9 | 1.21 | 2.7 |
|  | σ (n=3) | 1.04E+05 | 2.84E-04 | 1.47 | 5.5 | 1.7 | 2.1 |
| FGF18* | µ (n=3) | 2.70E+05 | 1.05E-03 | **4.17** | 36.2 | 0.57 | 2.3 |
|  | σ (n=3) | 8.84E+04 | 5.69E-04 | 2.71 | 24.8 | 0.62 | 1.5 |
| FGF4 | µ (n=3) | 4.11E+10 | 2.40E+01 | **0.27** | 22.8 | 3.63 | 72 |
|  | σ (n=3) | 6.96E+10 | 4.15E+01 | 0.29 | 10.4 | 1.97 | 39.8 |
| FGF5 | µ (n=3) | 6.68E+05 | 7.85E-03 | **917.01** | 29.7 | 2373.35 | 65 |
|  | σ (n=3) | 1.07E+06 | 6.29E-03 | 1573.56 | 46.6 | 3644.27 | 52 |
| FGF6 | µ (n=3) | 1.09E+10 | 3.33 | **0.37** | 10.3 | 0.78 | 45.3 |
|  | σ (n=3) | 1.89E+10 | 5.76 | 0.21 | 13.9 | 0.87 | 43.4 |
| FGF3 | µ (n=3) | 2.75E+05 | 1.34E-02 | **40.89** | 21421.7 | 461.3 | 95 |
|  | σ (n=3) | 2.45E+05 | 2.33E-02 | 70.77 | 31017.3 | 638.04 | 0 |
| FGF7 | µ (n=3) | 4.31E+04 | 3.53E-03 | **3365.36** | 3416.4 | 165.9 | 13.3 |
|  | σ (n=3) | 7.02E+04 | 2.52E-03 | 5029.77 | 3037 | 277.27 | 7.6 |
| FGF10 | µ (n=3) | 1.00E+05 | 1.99E-02 | **530.48** | 1832.3 | 9.51 | 39.7 |
|  | σ (n=3) | 1.08E+05 | 1.73E-02 | 597.92 | 2312 | 8.57 | 48 |
| FGF22 | (n=1) | 2.62E+01 | 3.89E-01 | **14840000** | 0.3 | 842 | 95 |
