# Supplementary figures and images for: In vitro and in vivo characterization of Recifercept, a soluble fibroblast growth factor receptor 3, as treatment for achondroplasia
Source: PLoS One. 2020 Dec 28;15(12):e0244368. doi: 10.1371/journal.pone.0244368 (PMC7769458; doi:10.1371/journal.pone.0244368)

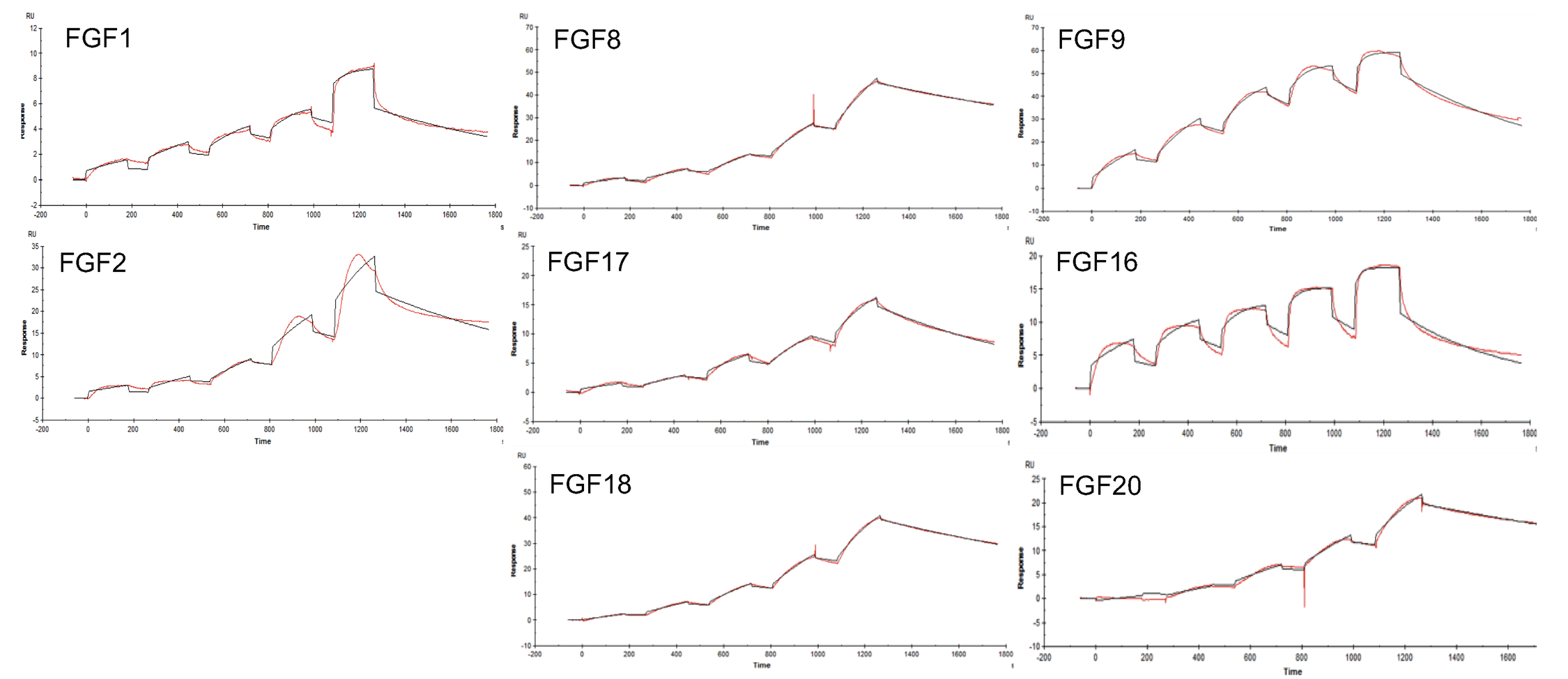

Supplement: S1 Fig — Binding between Recifercept and human FGFs isoforms was determined by surface plasmon resonance spectroscopy (SPR). Reference responses from the control Fc (blank immobilization), were subtracted from Recifercept Fc for each analyte injection. The resulting sensorgrams were used for kinetic parameter determination by globally fitting the entire association and dissociation phases to a 1: 1 interaction. hFGF9 was used as an internal run control and was loaded before and after the tested subfamilies. For each FGF, a single cycle kinetic was performed with 5 concentrations from 0 to 16 nM. (TIF) [file pone.0244368.s003.tif]

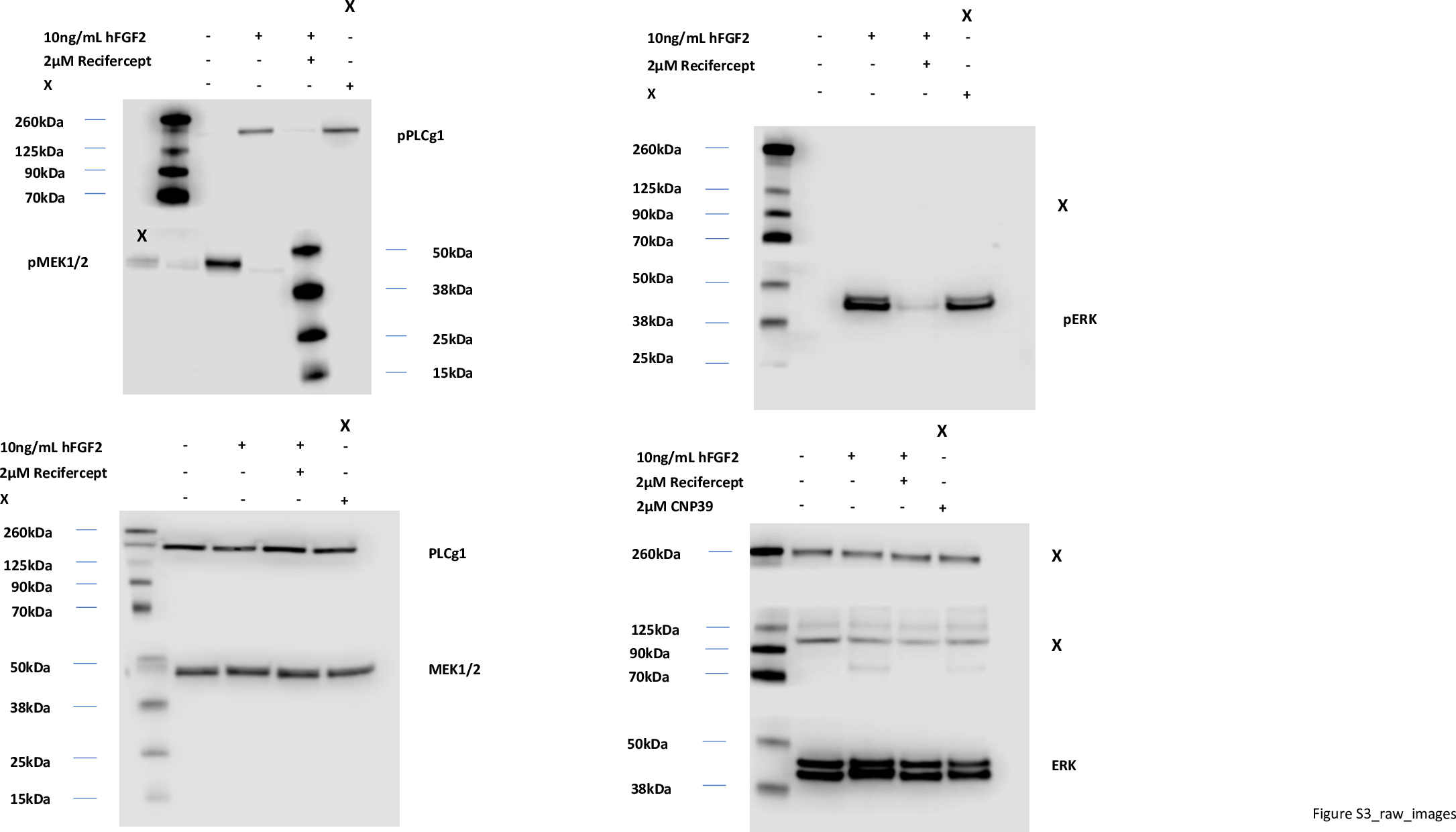

Supplement: S2 Fig — Western blots from Fig 3 were taken from these raw images. All lane marked with a “X” have not been taken into account for the present study. (TIF) [file pone.0244368.s004.tif]
